# A Targeted Approach to Improve Asthma Control Using Community Pharmacists

**DOI:** 10.3389/fphar.2021.798263

**Published:** 2021-12-27

**Authors:** Sarah Serhal, Bandana Saini, Sinthia Bosnic-Anticevich, Ines Krass, Lynne Emmerton, Bonnie Bereznicki, Luke Bereznicki, Bernadette Mitchell, Frances Wilson, Bronwen Wright, Kiara Wilson, Naomi Weier, Rebecca Segrott, Rhonda Cleveland, Stephen Jan, Sana Shan, Laurent Billot, Carol Armour

**Affiliations:** ^1^ Woolcock Institute of Medical Research, Sydney, NSW, Australia; ^2^ School of Pharmacy, The University of Sydney, Sydney, NSW, Australia; ^3^ Central Sydney Area Health Service, Sydney, NSW, Australia; ^4^ Curtin Medical School, Curtin University, Perth, WA, Australia; ^5^ Tasmanian School of Medicine, Hobart, TAS, Australia; ^6^ School of Pharmacy and Pharmacology, University of Tasmania, Hobart, TAS, Australia; ^7^ The Pharmaceutical Society of Australia, Deakin, ACT, Australia; ^8^ The Pharmacy Guild, Barton, ACT, Australia; ^9^ National Asthma Council Australia, South Melbourne, VIC, Australia; ^10^ The George Institute, Newtown, NSW, Australia; ^11^ Faculty of Medicine, University of New South Wales, Sydney, NSW, Australia

**Keywords:** asthma, community pharmacy, asthma control, implementation, pharmacy services, health services

## Abstract

**Background:** Building on lessons learnt from evidence-based community pharmacy asthma management models, a streamlined and technology supported Pharmacy Asthma Service (PAS) was developed to promote the integration of the service into routine practice.

**Objective:** This study investigates the efficacy of the PAS in improving asthma symptom control and other health outcomes.

**Methods:** A two-arm pragmatic cluster randomized controlled trial was implemented in 95 pharmacies across three Australian States. Participants were adults with poorly controlled asthma as per the Asthma Control Questionnaire (ACQ), with or without allergic rhinitis. Patients within the PAS arm engaged in four consultations with the pharmacist over a 12-month period. An evidence-based algorithm guided pharmacies, via a trial specific software, to deliver a series of interventions targeting three issues underpinning uncontrolled asthma (medication use and adherence, inhaler technique, and allergic rhinitis management) to patient clinical asthma status and patient need. Comparator arm patients received a minimal intervention likened to usual practice involving referral of eligible patients to the GP and two follow-up consultations with their pharmacist to collect comparative data.

**Results:** In total, 143 of 221 PAS patients (65%) and 111 of 160 comparator patients (69%) completed the trial. Improvements in asthma control were achieved in both the PAS (mean difference (MD) in ACQ from baseline = −1.10, *p* <.0001) and comparator (MD in ACQ from baseline = −0.94, *p* <.0001) arms at the trial end; however, there were no significant differences between the two arms (MD = −0.16, 95% CI −0.41 to 0.08, *p* = 0.19). Patients’ quality of life in the PAS arm improved significantly when compared with the comparator arm (MD in Impact of Asthma on Quality-of-Life Questionnaire (IAQLQ) = −0.52, 95% CI −0.89 to −0.14, *p* = 0.0079).

**Conclusion:** Despite the PAS achieving a greater improvement in patients’ quality of life, the pharmacist-led service and usual practice arm produced comparable improvements in asthma control. These results ask us to reflect on current standards of usual care, as it appears the standard of asthma care in usual practice has evolved beyond what is reported in the literature.

## Introduction

Optimal management of asthma is known to save lives; however, suboptimal asthma control within the community is globally evident and is often underpinned by ineffective use of effective medicines (Rabe et al., 2004; Price et al., 2015; Reddel et al., 2015; World Health Organisation, 2020). Consequently, asthma is responsible for approximately 1145 fatalities per day globally (World Health Organisation, 2020), the majority of which are considered preventable (Suissa et al., 2000; Peters et al., 2006; Global Initiative for Asthma, 2020). Although asthma management occurs fundamentally within primary care, (Qazi et al., 2021), in Australia it is estimated that people visit a pharmacy 18 times per year, (The Pharmacy Guild of Aus, 2019), and thus there is opportunity for pharmacists to add value to the care of asthma patients offered by general practitioners (GPs) and help mitigate current and future predicted asthma risk.

Research within Australia and elsewhere over the past two decades has demonstrated that structured pharmacy-based, pharmacist-delivered, patient-centered asthma management services can cost-effectively improve a range of patient outcomes (Saini et al., 2004; Gordois et al., 2007; Armour et al., 2013; Serhal et al., 2021). Despite demonstrated success in research, for a variety of reasons, including intervention complexity and the time required, these service models have unsuccessfully transitioned past novel interventions to become routinely embedded within community pharmacy practice.

In response to feedback from pharmacists in earlier trials (Armour et al., 2007; Gordois et al., 2007; Armour et al., 2013) and to increase accessibility of evidence-based interventions to asthma patients within the Australian community, a trial was designed to implement a streamlined and technologically supported Pharmacy Asthma Service (PAS). A new method of training was offered, which allowed pharmacists the flexibility to refine their knowledge and skills at a pace and time that suited their professional schedules (Serhal et al., 2021). Additionally, the service design aimed to reduce the cognitive and time burden on pharmacists by incorporating a novel, trial-specific, data collection software (Emmerton et al., 2012). As opposed to other studies, the PAS targeted only three evidence-based interventions known to improve control of asthma. These interventions addressed 1) poor adherence, (Reddel et al., 2015), characterized by underuse of preventer medication and/or overuse of reliever medication, 2) suboptimal inhaler technique, (Armour et al., 2011; Braido et al., 2016; Jahedi et al., 2017; Bosnic-Anticevich et al., 2018), and/or 3) uncontrolled allergic rhinitis (Price et al., 2005; Armour et al., 2011; Giavina-Bianchi et al., 2016; Price et al., 2016).

The objective of this study was to measure the relative efficacy of the PAS when compared to a minimal intervention (usual practice comparator) in a randomized controlled trial (RCT) design. The main outcome measured was asthma control.

## Methods

### Study Design

A cluster RCT design was used, with pharmacies the unit of cluster and patients the unit of analysis. All pharmacists and patients provided written or electronic informed consent. Recruitment commenced in July 2018 and the trial was completed in February 2020.

### Pharmacy Recruitment

Pharmacists from regional and metropolitan areas in New South Wales (NSW), Western Australia (WA), and Tasmania were invited to nominate their pharmacy via an online expression of interest form sent out by The Pharmacy Guild of Australia.

To participate, pharmacies were required to: be approved to dispense Pharmaceutical Benefits Scheme (PBS) medicines as part of the National Health Scheme defined in Section 90 of the National Health Act 1953 (Section 90 pharmacy); have an area physically separated from the retail trading floor to ensure privacy during consultations; and have a minimum of two pharmacists on duty at times when the service was to be delivered.

To ensure that rural and urban pharmacies were representative of the distribution of the Australian population in NSW, WA, and Tasmania, randomization was stratified according to State and remoteness index using the Pharmacy Access/Remoteness Index of Australia (PhARIA) (National Rural Health Alliance, 2011; The University of Adelaide Pharmacy ARIA PhARIA, 2019; The University of Adelaide. Hugo Centre for Migration and Population Research - Pharmacy ARIA PHARIA, 2019) and randomly assigned in a 1:1 ratio to PAS and comparator arms within each stratum by the investigative team statistician.

Pharmacists were offered remuneration for their participation, with PAS pharmacists receiving AU$120 per completed patient and comparator pharmacists receiving AU$35 per completed patient. These payments were considered compensation for professional time.

### Pharmacist Education

Prior to implementation, pharmacists in the PAS arm were required to pass both theoretical and skills-based training for assurance of the advanced clinical knowledge and skills required to deliver the PAS and compliance with the trial protocol (Serhal et al., 2021). Details of the education program have been published (Serhal et al., 2021). Pharmacists in the comparator arm required protocol training only.

### Patient Recruitment and Management

Upon completion of required training, pharmacies in both arms were asked to recruit a minimum of seven patients each. The sample size was based on feasibility established in previous studies, to account for predicted pharmacy and patient withdrawal rates, and the numbers required to show statistically significant change in asthma control (Saini et al., 2011; Serhal et al., 2021).

The primary inclusion criterion for patients was uncontrolled asthma as determined by a score ≥1.5 in the Asthma Control Questionnaire (ACQ) (Juniper et al., 2006). Additional criteria were age ≥18 years, ability to communicate with the pharmacist in English, regular patronage of the pharmacy, as assessed by the pharmacist (receiving medications from that pharmacy for the previous 12 months) and self-management of their medicines (as determined by the pharmacist).

Patients were excluded from the study if they had a high dependence on medical care (more than five morbidities and specialist care, or reliance on a caregiver), were unable to manage their own medicines (as determined by the pharmacist), and/or had a confirmed diagnosis of chronic obstructive pulmonary disorder (as reported by the patient) or a terminal illness.

**FIGURE 1:**
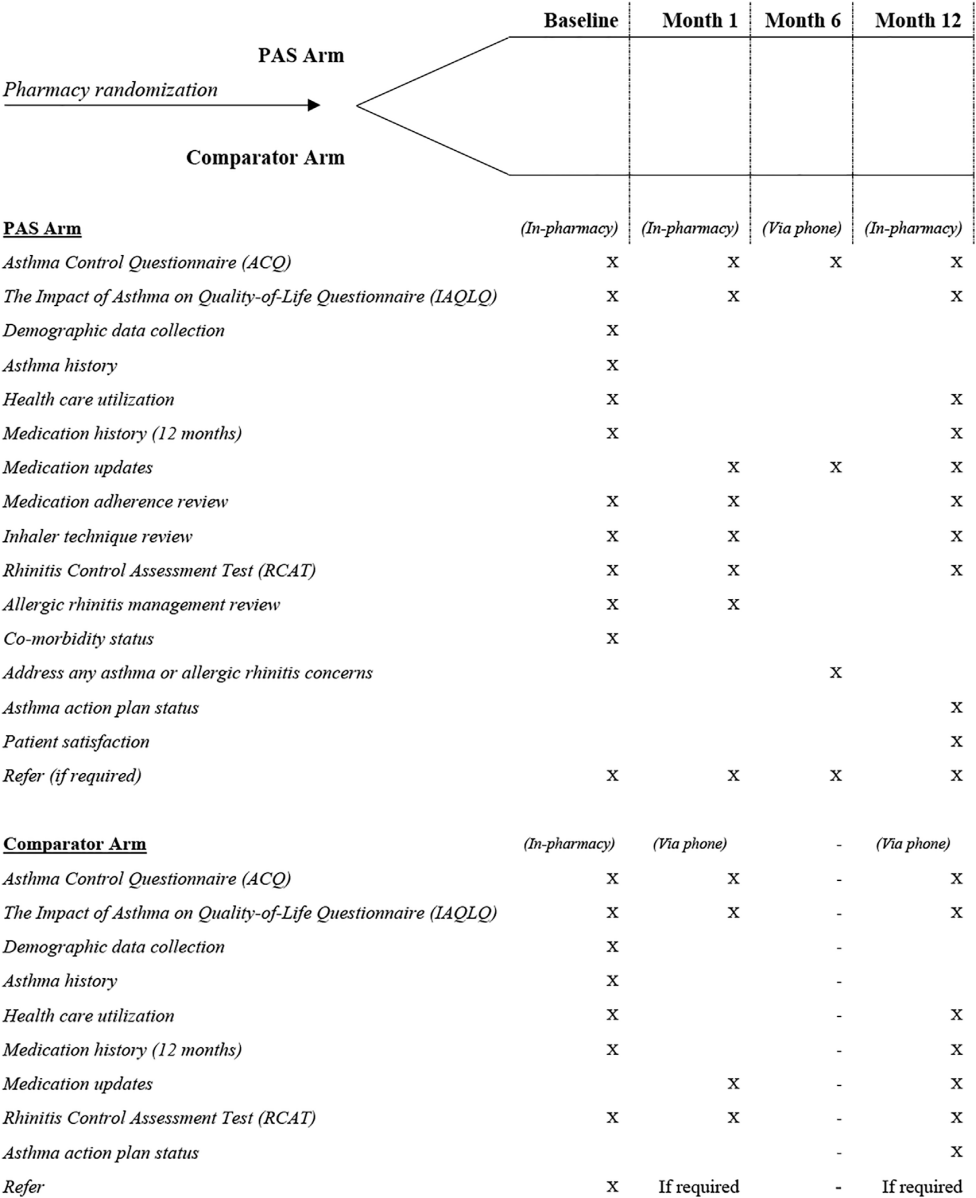
PAS and Comparator arm patient pathways.

Depending on the pharmacy in which they were recruited, patients proceeded into the PAS or comparator arm pathway. Figure 1 presents the protocol for each of the trial arms.

### PAS Protocol

The PAS was a pharmacist-led 12-month program conducted in the regular pharmacy of the recruited asthma patient. To deliver the service, the pharmacist undertook three private face-to-face consultations with the individual over a period of 12 months: at baseline, month 1 and month 12, with one additional telephone follow-up at month 6 to monitor progress and identify potential risks. After screening and identifying patients with uncontrolled asthma, patients underwent a baseline consultation consisting of education and counselling-based interventions centered on patient knowledge, beliefs of disease and medicines, and determining possible causes of poor control by addressing patient adherence, inhaler technique, and relevant co-morbidities such as allergic rhinitis. Pharmacists were also encouraged to refer patients with uncontrolled asthma control with unknown causes/complex issues to their GP.

### Comparator Arm

The comparator was a minimal intervention active control arm designed to mimic usual pharmacy practice whilst being structurally equivalent to the PAS arm for non-specific factors including format and data collection time points (Byrd-Bredbenner et al., 2017). Patients within the comparator arm were requested to attend three interactions with their pharmacist, the first (baseline) comprising an in-person session where asthma and allergic rhinitis control questionnaires were administered, and patients were given a referral to their GP. They were then contacted by the pharmacist by telephone one month and 12 months after baseline to collect comparative data (no interventions were made/undertaken).

### Data Collection

The project utilized GuildPath, a web-based, study-specific data collection software, to integrate data collection into routine pharmacy practice. GuildPath was integrated with GuildCare NG™, professional services software operating in over 5000 pharmacies in Australia (GuildLink, 2019). All validated questionnaires, visual analogue scales (VAS), checklists counselling and educational content were embedded into GuildPath’s underlying guiding algorithm. It was expected that pharmacists completed these questionnaires while engaging with the patient, using the tablet device provided for the trial. Records of each consultation were created automatically in each patient’s pharmacy GuildCare NG™ profile. Pharmacists were also able to generate personalized referral letters for the patient’s GP using a template embedded into GuildCare NG™.

Patient characteristics, including self-reported age of asthma symptom onset, smoking status, demographic data, including age, sex, location, education status and work status were collected at the beginning of the trial for patients in both arms.

### Outcome Measures

To evaluate the efficacy of the PAS relative to comparator arm, the following outcome measures were assessed.

#### Asthma Control

The primary outcome was asthma symptom control, as assessed via the ACQ (Juniper et al., 1999; Juniper et al., 2005) at all consultations including those conducted via telephone for patients in both arms of the study. A score of 1.5 or greater is considered an indication of uncontrolled asthma (Juniper et al., 2006). Additionally, patients who did not complete the full service were contacted at the end of the trial (when their final follow-up would have been due) to determine asthma control.

#### Quality of Life

Patient quality of life was evaluated via the Impact of Asthma on Quality of Life Questionnaire (IAQLQ) (Marks et al., 1992) at baseline, month 1 and month 12 for patients in both arms.

#### Healthcare Utilization

The self-reported number of hospitalizations and emergency department presentations in the preceding 12 months, and whether a patient recalled receiving a lung function test in the preceding 12 months, were recorded at baseline and month 12 for patients in both arms. Medicare Benefits Schedule (MBS)^1^ data were also collected for the duration of each patient’s involvement and 12 months prior, to evaluate the number of physician visits.

#### Medication Adherence

Preventer therapy adherence for each patient was assessed from PBS^2^ data spanning the 12 months preceding data collection and the 12 months they were involved in the trial. The data provided lists of all subsidized prescriptions collected by each patient over the given period (Australian Government Department of Health, 2021c). Adherence was calculated using the proportion of days covered (PDC) method (Raebel et al., 2013; National Center for Chronic Disease Prevention and Health Promotion, 2015; American Pharmacist Association. Measuring Adherence, 2020). A patient with a PDC of 80% or higher was considered adherent (Karve et al., 2009). Prescribed dosage information for each individual was not available in PBS data, so PDC calculations were based on standard dose. Standard dosage was based on the minimum effective adult dose required for each formulation/product, as recommended by the *Australia Medicines Handbook*, (Australian Medicines Handbook, 2020), *Therapeutic Guidelines* (Therapeutic Guidelines Limited eTG complete, 2019), and the *Australian Asthma Handbook* (National Asthma Council Australia, 2015). For the PAS arm, patients were also asked to self-assess their adherence using VAS at baseline and month 12 (Amico et al., 2006; McDowell, 2006; Nau et al., 2007; Ivanova et al., 2008).

#### Reliever Use

Data collected from ACQ Question 6 [*On average in the last week how many puffs of relief medication (short-acting bronchodilator such as Ventolin* ^*®*^ *, Bricanyl* ^*®*^ *etc.*) *have you used each day?*] (Juniper et al., 1999; Juniper et al., 2005) were used to assess patient reliever use, and evaluated at baseline and month 12. Reliever use was dichotomized as “appropriate use” (up to two inhalations most days in the preceding 7 days) and “overuse” (three or four inhalations or greater in the preceding 7 days) (Stanford et al., 2012). PAS arm patients were also asked to self-report the number of times they had used their reliever in the past 7 days and the number of puffs required on each occasion to obtain relief. All measures used to determine reliever use were based on self-report as opposed to pharmacy dispensing data, as in Australia, reliever inhalers are scheduled as Pharmacist Only Medicines, which means reliever inhalers may be obtained over-the-counter with no requirement for the transaction to be recorded. The only circumstance in which reliever supply would be recorded is when the patient is eligible for a healthcare subsidy/concession (with inhalers dispensed via prescription at a reduced price) which would only account for a proportion of the sample.

#### Inhaler Technique Competency (PAS Arm Only)

Inhaler technique assessment was conducted by observing the patient demonstrate use of their inhaler(s)/device(s), against National Asthma Council Australia device-specific checklists (National Asthma Council Australia, 2016). Patients who were not able to correctly use their inhaler(s) on their first attempt had a physical demonstration by the pharmacist with a placebo inhaler and the patient was asked to demonstrate again until device mastery was achieved (up to a maximum of three times). Inhaler technique was assessed at each in-person consultation.

#### Allergic Rhinitis Control

Patients in both arms who had comorbid allergic rhinitis completed the Rhinitis Control Assessment Test (RCAT) at baseline, month 1 and month 12 (Schatz et al., 2010). Based on the RCAT score, patients scoring ≤ 21 were considered clinically “symptom uncontrolled”, while those scoring >21 were considered “symptom controlled”.

#### Asthma Action Plan Possession

Asthma action plan possession was recorded only upon completion of the trial at month 12 in both PAS and comparator arms. Pharmacists were asked to refer patients without a current plan to their GP.

### Data Analysis

A sample size of 80 pharmacies (40 per arm), each recruiting seven patients (total sample size = 560 patients) had 90% power to detect a 20% absolute improvement in the proportion of patients with controlled asthma at 12 months, assuming that no more than 30% would have controlled asthma in the comparator group. This assumed an intra-cluster correlation of 0.1 and allowed for 20 and 15% of patient and pharmacy withdrawal, respectively.

The proportion of patients who had controlled asthma at 12 months were analyzed using a repeated measure mixed logistic regression including both the month 1 and month 12 values as dependent variables. Fixed effects included the random group allocation, the visit (month 1 or month 12), the interaction between the arm allocation and the visit and the baseline value of the ACQ score as a continuous variable. A random intercept per cluster was included to account for intra-cluster correlation. The main effect of the PAS was estimated as the odds-ratio (PAS vs comparator) together with its 95% confidence interval. The model was rerun after adjusting for the following baseline patient covariates: age, whether the patient had a lung function test within 12 months of baseline, smoking status, work status and presence of co-morbid allergic rhinitis. Missing data for ACQ scores at month 1 and month 12 were imputed using a multiple imputation technique (fully conditional specification with predictive mean matching) as part of the sensitivity analysis. The imputation model included random group allocation, the visit (month 1 or month 12), baseline value of the ACQ score and cluster variable along with the following baseline variables: IAQLQ score, age, sex, work status, education status, age since diagnosis, history of lung function test, smoking status and allergic rhinitis status. A subgroup analysis was also performed by adding a subgroup variable as well as its interaction term to the model used for the primary outcome analysis. Following variables were analyzed: Age (<56, ≥56 years), sex, pharmacy state and pharmacy remoteness.

Dichotomous secondary outcomes were analyzed using the same method as for the primary outcome, while continuous secondary outcomes were analyzed using a similar approach with a linear model instead of a logistic one. No adjusted or subgroup analyses were applied to the secondary outcomes.

All data pertaining to a trial consultation, regardless of the exact timing in which the consultation was conducted, was included in the analysis. To account for variability in timing in which some consultations were conducted, a sensitivity analysis was performed. The sensitivity analysis involved re-running the primary outcome analysis model including only sessions conducted within the following time frames:

i) Month 1–20 to 50 days included;
ii) Month 6–160 to 220 days included; and
iii) Month 12–330 to 420 days included.

Individual participants were excluded from the sensitivity analysis if their visits fell outside the specified time windows.

Analyses were conducted on an intention-to-treat basis. A significance level of *p* <0.05 was used for all statistical procedures. Process variables were computed in SPSS Version 25. Analyses were performed primarily using SAS software (Version 9.4; SAS Institute) in accordance with the pre-determined statistical analysis plan (Billot et al., 2020).

### Governance

This trial was approved by the Human Research Ethics Committees of The University of Sydney, Curtin University and The University of Tasmania and funded by the Australian Government Department of Health via the 6th Community Pharmacy Agreement (Australian New Zealand Clinical Trials Registry, 2018). The trial is registered in the Australian New Zealand Clinical Trials Registry (ACTRN12618000313235) and was designed and implemented by a consortium led by the Woolcock Institute of Medical Research. Members of the implementation consortium included The University of Sydney, Curtin University, University of Tasmania, The Pharmacy Guild of Australia, Pharmaceutical Society of Australia, The National Asthma Council Australia and The George Institute (Australian New Zealand Clinical Trials Registry, 2018).

## Results

### Process

In total, 95 community pharmacies (51 PAS and 44 comparator) across NSW (*n* = 63), WA (*n* = 21) and Tasmania (*n* = 11) participated and recruited 381 patients into the trial. The mean number of patients recruited per pharmacy was four, ranging from one to 16 patients per pharmacy. Sixteen pharmacies (17%) withdrew from the study after recruiting patients into the trial (nine PAS pharmacies and seven comparator pharmacies).

Both PAS and comparator arm patients were comparable at baseline (Table 1). Most patients were female (69.6%),non-smokers(86.4%), and with self-reported allergic rhinitis (71%). Thirty-three percent of the cohort were retired, 48% had tertiary qualifications and 45% had asthma as a child.

**TABLE 1:** Patient characteristics.

	PAS	Comparator	Total	*p*-value
Pharmacy state	*n* = 221	*n* = 160	*n* = 381	0.6502
NSW	159 (71.9%)	113 (70.6%)	272 (71.4%)	
WA	40 (18.1%)	25 (15.6%)	65 (17.1%)	
Tasmania	22 (10.0%)	22 (13.8%)	44 (11.5%)	
Pharmacy remoteness^a^	*n* = 221	*n* = 160	*n* = 381	0.2886
Highly Accessible	143 (64.7%)	110 (68.8%)	253 (66.4%)	
Accessible	59 (26.7%)	29 (18.1%)	88 (23.1%)	
Moderately Accessible, Remote, Very remote	19 (8.6%)	21 (13.1%)	40 (10.5%)	
Age (years)	*n* = 221	*n* = 160	*n* = 381	0.2896
18 to 25	10 (4.5%)	14 (8.8%)	24 (6.3%)	
26 to 35	23 (10.4%)	12 (7.5%)	35 (9.2%)	
36 to 45	45 (20.4%)	13 (8.1%)	58 (15.2%)	
46 to 55	34 (15.4%)	25 (15.6%)	59 (15.5%)	
>55	109 (49.3%)	96 (60.0%)	205 (53.8%)	
Sex	*n* = 221	*n* = 160	*n* = 381	0.6066
Male	65 (29.4%)	51 (31.9%)	116 (30.4%)	
Female	156 (70.6%)	109 (68.1%)	265 (69.6%)	
Work situation	*n* = 221	*n* = 160	*n* = 381	0.2090
Full-time employed	56 (25.3%)	34 (21.3%)	90 (23.6%)	
Home duties	12 (5.4%)	21 (13.1%)	33 (8.7%)	
Part time or casually employed	53 (24.0%)	29 (18.1%)	82 (21.5%)	
Retired/pensioner	75 (33.9%)	52 (32.5%)	127 (33.3%)	
Unemployed or seeking work	10 (4.5%)	13 (8.1%)	23 (6.0%)	
Full-time carer	5 (2.3%)	2 (1.3%)	7 (1.8%)	
Other	10 (4.5%)	9 (5.6%)	19 (5.0%)	
Level of education	*n* = 221	*n* = 160	*n* = 381	0.9749
No formal education	3 (1.4%)	4 (2.5%)	7 (1.8%)	
Primary school	7 (3.2%)	4 (2.5%)	11 (2.9%)	
High school	101 (45.7%)	81 (50.6%)	182 (47.8%)	
Tertiary non-university (e.g., TAFE)	61 (27.6%)	35 (21.9%)	96 (25.2%)	
University	39 (17.6%)	31 (19.4%)	70 (18.4%)	
Postgraduate	10 (4.5%)	5 (3.1%)	15 (3.9%)	
Age at asthma onset	*n* = 221	*n* = 160	*n* = 381	0.7374
0–5 years	49 (22.2%)	41 (25.6%)	90 (23.6%)	
6–15 years	52 (23.5%)	28 (17.5%)	80 (21.0%)	
16–34 years	57 (25.8%)	40 (25.0%)	97 (25.5%)	
35–55 years	36 (16.3%)	31 (19.4%)	67 (17.6%)	
>55 years	27 (12.2%)	20 (12.5%)	47 (12.3%)	
Ever had a lung function test	*n* = 221	*n* = 160	*n* = 381	0.0514
No	54 (24.4%)	54 (33.8%)	108 (28.3%)	
Yes	167 (75.6%)	106 (66.3%)	273 (71.7%)	
Last lung function test	*n* = 167	*n* = 106	*n* = 273	0.4040
<12 months ago,	58 (34.7%)	41 (38.7%)	99 (36.3%)	
≥12 months ago,	109 (65.3%)	65 (61.3%)	174 (63.7%)	
Active smoker	*n* = 221	*n* = 160	*n* = 381	0.3812
No	194 (87.8%)	135 (84.4%)	329 (86.4%)	
Yes	27 (12.2%)	25 (15.6%)	52 (13.6%)	
History of hay fever	*n* = 221	*n* = 160	*n* = 381	0.3121
No	60 (27.1%)	49 (30.6%)	109 (28.6%)	
Yes	161 (72.9%)	111 (69.4%)	272 (71.4%)	
RCAT score^b^	*n* = 221	*n* = 160	*n* = 381	0.2360
Median (Q1; Q3)	21.0 (16.0; 25.0)	20.0 (16.0; 24.0)	20.0 (16.0; 25.0)	
IAQLQ score^c^	*n* = 221	*n* = 160	*n* = 381	0.3747
Median (Q1; Q3)	3.3 (2.0; 4.9)	3.1 (1.5; 4.4)	3.1 (1.8; 4.8)	
ACQ score^d^	*n* = 221	*n* = 160	*n* = 381	0.8105
Median (Q1; Q3)	2.3 (1.8; 3.0)	2.2 (1.7; 2.8)	2.2 (1.7; 3.0)	

Note: All baseline measures unless recorded otherwise.

a Participating pharmacies were identified as either highly accessible (PhARIA Category 1), accessible (PhARIA Categories 2 and 3) or moderately accessible, remote, and very remote (PhARIA Categories 4, 5, and 6) (National Rural Health Alliance, 2011; The University of Adelaide Pharmacy ARIA PhARIA, 2019; The University of Adelaide. Hugo Centre for Migration and Population Research - Pharmacy ARIA PHARIA, 2019).

b Rhinitis Control Assessment Test (RCAT) scores lie between 6 and 30. The lower the score, the more severe the allergic rhinitis; the higher the score, the less severe the allergic rhinitis. Patients scoring ≤21 are considered clinically “symptom uncontrolled”; those scoring >21 are considered “symptom controlled” (Meltzer et al., 2013).

c The Impact of Asthma on Quality of Life Questionnaire (IAQLQ) scores lie between 0 and 10. Higher scores represent a greater impact of asthma on quality of life. (Marks et al., 1992).

d Asthma Control Questionnaire (ACQ) score lies between 0 (totally controlled) and 6 (extremely poorly controlled). A score of 1.5 or greater is considered an indication of poorly controlled asthma. (Juniper et al., 2006).

**FIGURE 2:**
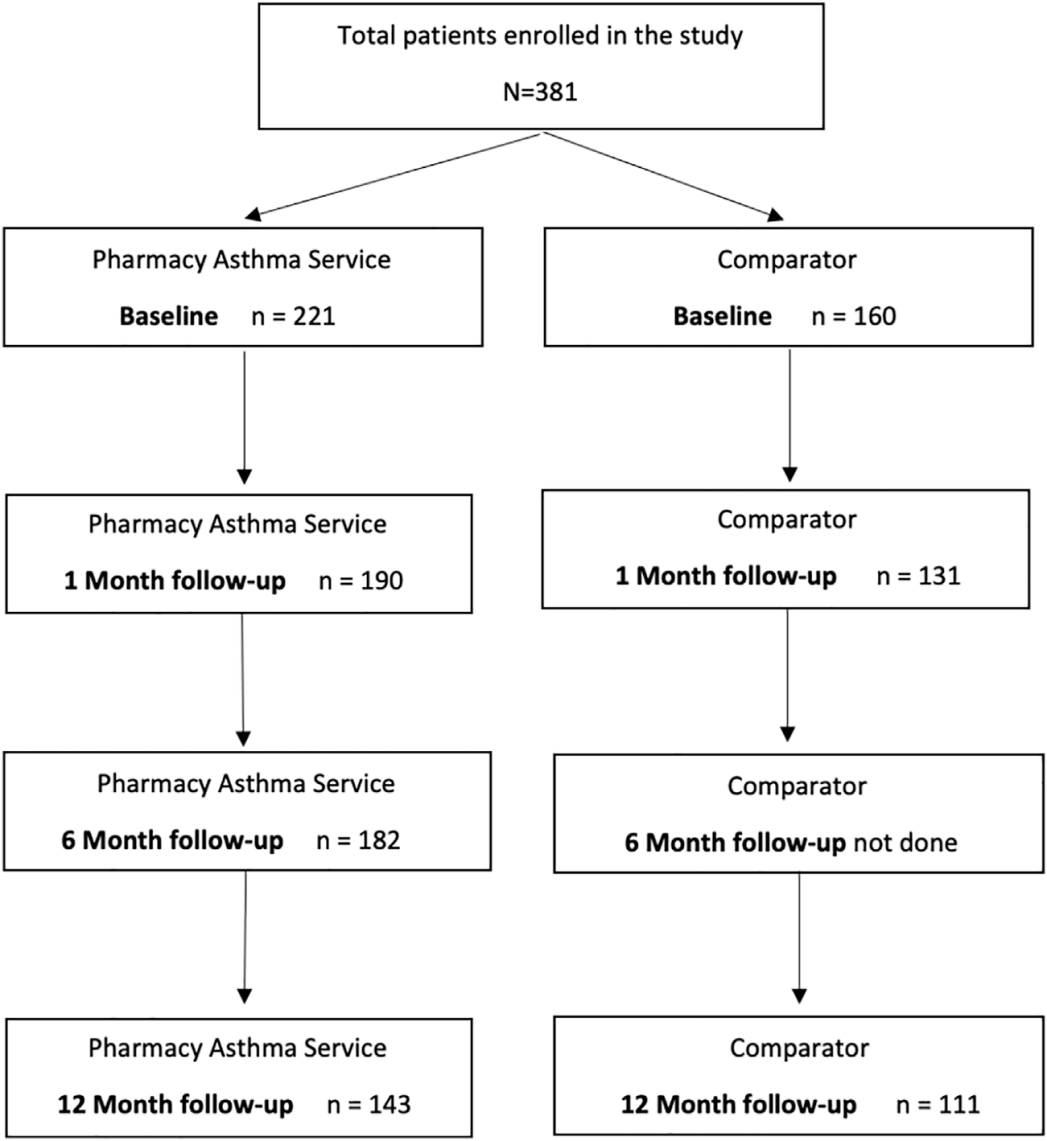
Patient Consort Diagram.

In total, 254 patients (143 PAS arm and 111 comparator arm) completed the trial by attending all consultations during the 12-months. Patient engagement throughout the trial is depicted in Figure 2. A total of 127 (33%) patients did not complete the full 12-month trial (78 PAS arm patients and 49 comparator arm patients). Reasons for non-completion included the patient being too busy (16%), no longer willing to participate (14%), unwell (10%) or having relocated (8%) as well as the pharmacy’s inability to contact the patient (43%) or unwillingness or inability to complete consultations (i.e., the pharmacy had been sold, or no trained pharmacist remained employed) (21%).

Of the non-completing patients, 41.7% were successfully contacted at month 12. The mean ACQ score reported by these patients at month 12 was 1.7 (±1.0), and 60.4% had uncontrolled asthma at that point in time, as indicated by their ACQ score. When patients who completed the full 12-month trial were compared to those who did not, the two groups were overall quite comparable; however, those who did not complete were less likely to have a history of allergic rhinitis (*p* = 0.0050), their asthma impacted their quality of life negatively to a greater extent (*p* = 0.0004), had poorer asthma control (*p* = 0.0029), and were recruited from highly accessible metropolitan pharmacies (*p* <0.001) and from NSW (*p* = 0.0089).

A total of 736 PAS sessions were conducted over the 12-month period, with a median number of 12 sessions conducted per pharmacy, ranging from zero to 58. In total, the comparator pharmacies conducted 402 sessions over the 12-month period, with a median of 12 sessions per pharmacy, ranging from zero to 21.

On average, it took PAS arm pharmacists just under 100 min to deliver the full 12-month intervention per patient; this ranged from 32 to 225 min. For comparator pharmacists, it took on average 55 min to deliver the minimal intervention over 12 months per patient; this ranged from 18 to 115 min.

### Outcomes

A summary of primary and secondary outcomes is presented in Table 2.

**TABLE 2:** Primary and secondary outcomes.

	PAS Mean (SE) or n (%)	Comparator Mean (SE) or n (%)	Mean difference or odds ratio (95%CI)	p-value
Proportion with ACQ Score^a^ <1.5 (primary analysis)				
Baseline	0 (0.0)	0 (0.0)	—	—
Month 1	85 (44.7)	72 (55.0)	0.67 (0.40 to 1.13)	0.1300
Month 12^e^	88 (61.5)	59 (53.2)	1.51 (0.84 to 2.70)	0.1669
ACQ score^a^				
Month 1	1.58 (0.07)	1.58 (0.09)	0.00 (−0.22 to 0.23)	0.9736
Baseline to month 1	−0.86 (0.07)	−0.86 (0.09)	—	—
p-value	<.0001*	<.0001*	—	—
Month 12	1.34 (0.08)	1.50 (0.09)	−0.16 (−0.41 to 0.08)	0.1960
Baseline to month 12	−1.10 (0.08)	−0.94 (0.09)	—	—
p-value	<0.0001*	<0.0001*	—	—
IAQLQ score^b^				
Baseline	3.5 (1.9)	3.2 (2.0)	—	—
Month 1	2.25 (0.11)	2.45 (0.14)	−0.20 (−0.55 to 0.15)	0.2667
Baseline to month 1	−0.97 (0.11)	−0.77 (0.14)	—	—
p-value	<0.0001*	<0.0001*	—	—
Month 12^e^	1.94 (0.13)	2.45 (0.14)	−0.52 (−0.89 to −0.14)	0.0079^b^
Baseline to month 12	−1.28 (0.13)	−0.077 (0.14)	—	—
p-value	<0.0001*	<0.0001*	—	—
RCAT score^c^				
Baseline	20.8 (5.4)	19.9 (5.1)	—	—
Month 1	22.61 (0.40)	21.94 (0.48)	0.67 (-0.57 to 1.91)	0.2866
Baseline to month 1	2.36 (0.40)	1.69 (0.48)	—	—
p-value	<0.0001*	0.0006*	—	—
Month 12^e^	22.04 (0.44)	21.54 (0.51)	0.50 (-0.84 to 1.83)	0.4640
Baseline to month 12	1.79 (0.44)	1.30 (0.51)	—	—
p-value	<.0001*	0.0122*	—	—
Number of emergency department presentations for asthma				
Baseline	0.5 (2.21)	0.5 (1.36)	—	—
Month 12^e^	0.1 (0.49)	0.3 (0.76)	0.18 (−0.01; 0.37)	0.0620
p-value	0.0115*	0.2470	—	—
Number of hospital admissions for asthma				
Baseline	0.3 (0.95)	0.4 (1.35)	—	—
Month 12^e^	0.1 (0.45)	0.3 (0.81)	0.20 (−0.00; 0.404)	0.0532
p-value	0.0519	0.4585	—	—
Number of GP visits^d^				
Baseline	20.5 (20.87)	17.4 (14.84)	—	—
Month 12^e^	22.3 (22.82)	24.2 (20.11)	2.56 (−1.17; 6.292)	0.1770
p-value	0.1323	0.0110*	—	—
Adherence				
Baseline	58/108 (53.7%)	53/81 (65.4%)	—	—
Month 12	54/108 (50.0%)	41/81 (50.6%)	1.08 (0.52, 2.24)	0.8375

*Significant result.

a Asthma Control Questionnaire (ACQ) score lies between 0 (totally controlled) and 6 (extremely poorly controlled). A score of 1.5 or greater is considered an indication of poorly controlled asthma (Juniper et al., 2006).

b The Impact of Asthma on Quality of Life Questionnaire (IAQLQ) scores lie between 0 and 10. Higher scores represent a greater impact of asthma on quality of life (Marks et al., 1992).

c Rhinitis Control Assessment Test (RCAT) scores lie between 6 and 30. The lower the score, the more severe the allergic rhinitis; the higher the score, the less severe the allergic rhinitis. Patients scoring ≤ 21 are considered clinically “symptom uncontrolled”; those scoring > 21 are considered “symptom controlled” (Meltzer et al., 2013).

d GP visits for asthma were determined using Medicare Benefits Schedule data for each patient.

e Including only those randomized patients who also have 12 months follow-up data.

#### Asthma Control

**FIGURE 3:**
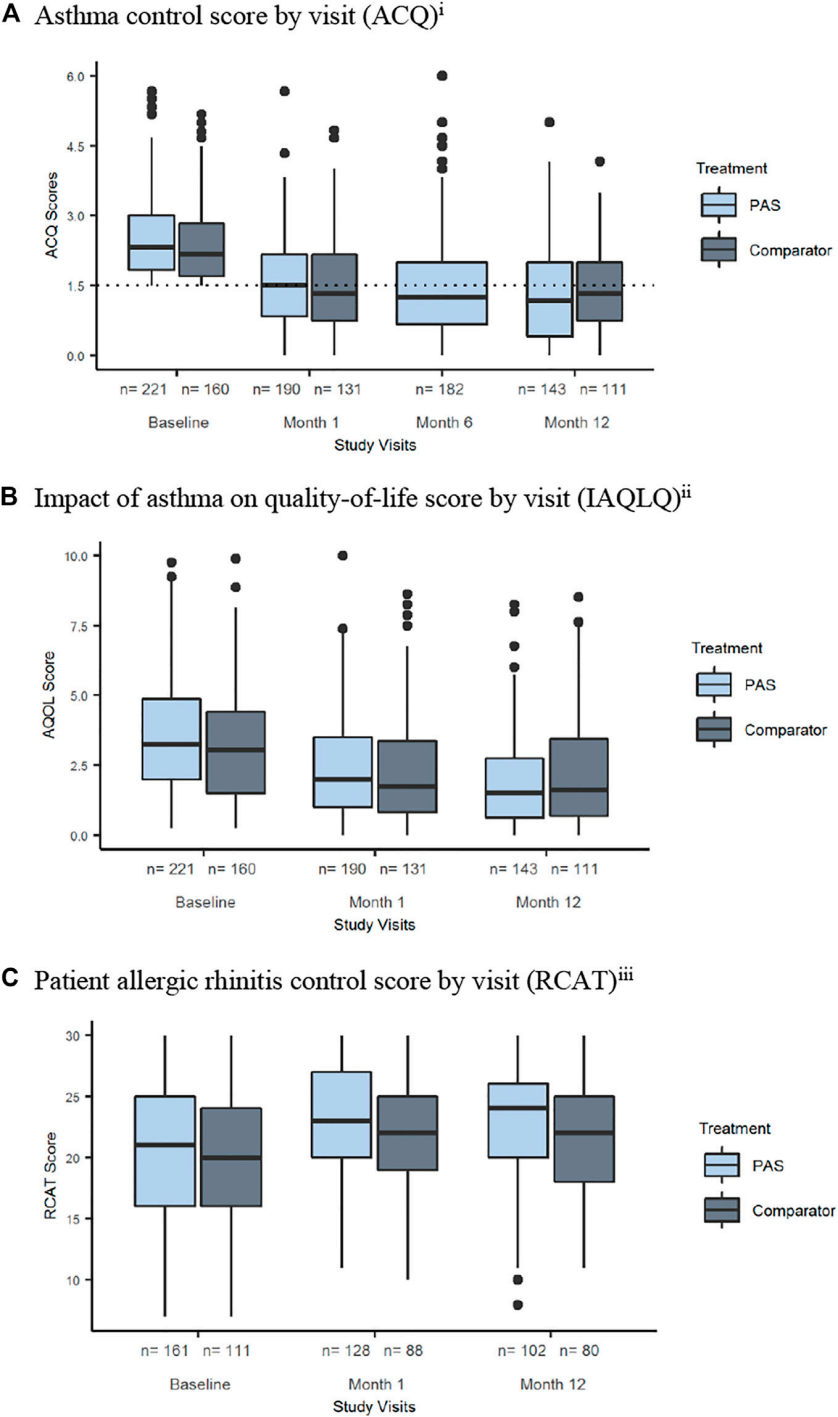
Primary and secondary outcomes. **(A)** Asthma control score by visit (ACQ)^i^. **(B)** Impact of asthma on quality-of-life score by visit (IAQLQ)^ii^. **(C)** Patient allergic rhinitis control score by visit (RCAT)^iii^. Note: i) Asthma Control Questionnaire (ACQ) score lies between 0 (totally controlled) and 6 (extremely poorly controlled). A score of 1.5 or greater is considered an indication of poorly controlled asthma (Juniper et al., 2006) Change in score of 0.5 is considered a clinically significant change (Juniper et al., 2006) Note no assessment of ACQ at month 6 in comparator arm. ii) The Impact of Asthma on Quality of Life Questionnaire (IAQLQ) scores lie between 0 and 10. Higher scores represent a greater impact of asthma on quality of life (Marks et al., 1992). iii) Rhinitis Control Assessment Test (RCAT) Scores lie between 6 and 30. The lower the score, the more severe the allergic rhinitis; the higher the score, the less severe the allergic rhinitis. Patients scoring ≤21 are considered clinically “symptom uncontrolled”; those scoring >21 were considered “symptom controlled” (Meltzer et al., 2013).

Asthma control significantly improved over the 12-month period of the trial in both the PAS (*p* <.001) and comparator arms (*p* <.001) (Figure 3A). However, the proportion of patients with controlled asthma at 12 months was not statistically different between the two arms (OR 1.51, 95% CI 0.84 to 2.70, *p* = 0.1669). Results from the sensitivity analyses involving analysis on restricted timeframes, missing data imputation as well as the subgroup analyses were consistent with the main analysis.

#### Quality of Life

Asthma quality of life scores improved significantly in the PAS arm when compared to the comparator arm (MD = −0.52, 95% CI −0.89 to −0.14, *p* = 0.0079) (Figure 3B).

#### Health Care Utilization

In the PAS arm, there was a significant decrease in the mean number of self-reported emergency department presentations during the 12 months of the trial compared with the 12 months prior to the trial (*p* = 0.0115). There was a significant increase in GP visits in the comparator arm during the trial (*p* = 0.0110). There were no significant differences between self-reported emergency department visits (*p* = 0.0620), hospital admissions (*p* = 0.0532) or MBS recorded GP visits (*p* = 0.1770) when the two arms were compared.

#### Medication Adherence

Of the total sample (*n* = 381), 378 patients consented to the collection of their PBS data, and 345 were able to be linked to complete PBS data collection: 205 (93%) patients from the PAS arm and 140 (86%) from the comparator arm. For patients who had their study data successfully paired with PBS data 12 months prior to baseline as well as 12 months during the trial, there were no significant differences (OR 1.08, 95% CI 0.52 to 2.24, *p* = 0.8375) when comparing adherence between the two trial arms at month 12.

Using patient *self-report*, patients reported using their preventer/controller medications on average 69% of the time in the 7 days preceding baseline, and this increased significantly to 76% in the 7 days preceding the patient’s month 12 consultation (*p* = 0.04).

#### Reliever Use

Self-reported reliever use in the preceding 7 days decreased significantly over time when figures were compared in the PAS arm (*p* = 0.034) and the comparator arm (*p* = 0.009) (Table 3) with no difference between the arms at month 12 (*p* = 0.3872).

**TABLE 3:** Patient reliever use.

		PAS *n* (%)	Comparator *n* (%)	Total	p-value
		*n* = 221	*n* = 160	*n* = 381	—
Baseline^a^	≤1–2 puffs/inhalations most days	55 (24.9)	59 (36.9)	114 (29.9)	0.1646
	≥3–4 puffs/inhalations most days	166 (75.1)	101 (63.1)	267 (70.1)	
		*n* = 143	*n* = 111	*n* = 254	
Month 12^a^	≤1–2 puffs/inhalations most days	91 (63.6)	63 (56.8)	154 (60.6)	0.3872
	≥3–4 puffs/inhalations most days	52 (26.4)	48 (43.2)	100 (39.4)	
p-value	—	0.034*	0.009*	—	—

* Note: Significant result.

a Based on patient responses to Q6 of the Asthma Control Questionnaire (ACQ). Number of puffs of reliever medication each day on average. The data were analysed using the binary comparison between up to 2 puffs (appropriate use) versus 3-4 puffs or greater (overuse).

PAS patients reported that in the 7 days prior to baseline, they used their reliever on average 15 times (ranging from zero to 140 times in that week). There was a significant reduction in self-reported reliever use by the end of the intervention; PAS patients reported using their reliever inhalers on average nine times in the 7 days preceding month 12 consultation (*p* = 0.0035). Additionally, the number of puffs required to obtain relief decreased amongst PAS patients over the 7 days preceding baseline (three puffs) to the 7 days preceding month 12 consultations (2 puffs) (*p* <.0001).

#### Inhaler Technique Competency (PAS Arm Only)

Exploration of the proportion of PAS patients who had device mastery at baseline prior to training, indicated 34, 40, and 46% of patients using pressurized metered-dose inhalers (pMDIs), dry-powder inhalers, and soft-mist inhaler devices, respectively, had mastery. Almost all patients using a pMDI (97%) and a dry-powder inhaler (97%) achieved device mastery after training (baseline), and mastery was sustained by over half the patients by month 12 (52% for pMDI and 72% for dry powder inhaler).

For patients using a soft-mist inhaler, device mastery was achieved by all and was sustained by 67% of patients by month 12; however, numbers were small (*n* = 12).

#### Allergic Rhinitis Control

In the PAS arm, 73% of patients self-reported having allergic rhinitis. Similarly, in the comparator arm, 69% of patients reported having allergic rhinitis. Significant improvement in allergic rhinitis control over time was recorded in both the PAS (*p* <.0001) and comparator arms (*p* = 0.0122) (Figure 3C). However, improvements in allergic rhinitis control were not significantly different upon comparison of the two arms.

Regarding allergic rhinitis management, at baseline, 86% of patients with allergic rhinitis in the PAS arm accepted a new recommendation by the pharmacist to help manage their allergic rhinitis (there were none prescribed for the comparator arm). Compared to baseline (50%), a higher proportion of patients were treating their allergic rhinitis symptoms at month 1 (66%). Only 42.0% of patients were taking the first-line recommended treatment at baseline (intranasal corticosteroid) (Bosnic-Anticevich et al., 2019). This improved marginally at month 1 to 55.3% (not statistically significant, *p* = 0.862). Oral antihistamines, although not recommended for first-line treatment, were the patient’s treatment of choice with 84% reporting use at baseline. This significantly decreased to 64% at month 1 (*p* = 0.003).

#### Asthma Action Plan Possession

At month 12, 39% of PAS patients and 49% of comparator patients were in possession of a current Asthma Action Plan. This difference was not significant (*p* = 0.20).

## Discussion

The PAS trial demonstrated that the integration of a structured evidence-based pharmacist-delivered service aimed at improving asthma care within community pharmacies achieved improvements in asthma control, allergic rhinitis control, reliever use, and health care utilization. The comparator arm also achieved similar improvements. However, the PAS significantly improved patients’ quality of life when compared to a minimal intervention comparator arm.

Patients who participated in the PAS also experienced significant improvements in *self-reported* adherence and inhaler technique over time.

The trial demonstrated that a significant improvement in asthma control for people with uncontrolled asthma was possible over a 12-month period, with a significant increase in the proportion of PAS and comparator arm patients experiencing good asthma control (ACQ score <1.5) (Juniper et al., 2006) at the trial’s end. These results, although surprising when compared to the parent trials upon which the study was designed, (Armour et al., 2007; Armour et al., 2013), are in line with clinical outcomes achieved in other interventions that have trialed similar services in community pharmacy settings (Schulz et al., 2001). The degree of improvement in asthma symptoms in both arms during the trial suggests that the act of identifying people with uncontrolled asthma with a series of validated questions serves as an important trigger for community pharmacists to implement strategies to improve asthma control. In addition, patients in the comparator arm who took up the recommended initial referral to the GP would have received GP care for their asthma which may explain the improvements observed in this group.

Literature shows that patient expectations of their pharmacists change after having participated in a pharmacy-based health service (Anderson et al., 2004; NaikPanvelkar et al., 2010). Thus, increasing community pharmacy health services combined with higher patient expectations may have led to an upwards shift in pharmacist skillsets and standards of practice. In this context, the identification of those with sub-optimal asthma or allergic rhinitis management may have propelled some comparator pharmacists to intervene beyond that specified in the trial protocol and beyond what the literature expects of usual care (Schneider et al., 2009). Indeed, the time taken to deliver the comparator protocol would suggest that more than the “standard” service was being delivered. The professional freedoms or heterogeneity amongst the comparator arm pharmacists may have compromised the fidelity of the comparator arm (Byrd-Bredbenner et al., 2017). Whether this occurred warrants further investigation.

Asthma is a chronic condition that cannot be cured, so steps towards minimizing patient burden, enhancing their emotional well-being, and improving their ability to wholly participate in work, social and school life unrestricted by asthma are key (Kheir et al., 2001; Kheir et al., 2004). Humanistic measures such as assessing asthma-related quality of life allow us to determine the personal significance of clinical improvements (Kheir et al., 2001). Literature has shown that patients’ experience of their disease can differ significantly to the health system standard measures that seek to define their condition (Boulet et al., 2002; Rabe et al., 2004; Fletcher and Hiles, 2013; Price et al., 2014). This study demonstrated a significant improvement in the quality of life for patients receiving the PAS. It may be that patients were empowered by the education and counselling to better manage the burden of their condition (Schulz et al., 2001) However, this warrants further investigation.

There was no significant improvement in patient adherence to preventer medication in PAS or comparator arms during the trial when measured using PBS records. At month 12, in both the PAS and comparator arms, adherence was approximately 50%, which suggests only half the patients were having their asthma medications dispensed at appropriate intervals. Despite the known benefits of regular preventer use on symptomatic control of asthma and reducing long term risks, asthma patients are known to not take their preventer therapy regularly (Ponieman et al., 2009; Boulet et al., 2012; Global Initiative for Asthma, 2018; Reddel et al., 2018; Riley et al., 2021). Rather, many people with asthma rely on reliever medications that provide immediate symptomatic relief and can be purchased without a prescription, at a lower price compared to preventer medicines in Australian pharmacies (Reddel et al., 2015). The high prevalence of poor adherence to preventer therapy is consistent with international studies, despite variations in thresholds and measurements used to classify adherence (Ponieman et al., 2009; Boulet et al., 2012; Global Initiative for Asthma, 2018; Reddel et al., 2018; Riley et al., 2021). In contrast, results from patient *self-reported* adherence, measured in the PAS arm, showed significant improvements in the use of their asthma medications, including an increase in preventer use and a decrease in reliever use over the 12 months of the study. This is not surprising, as patients are likely to report to the pharmacist that they are using their medications. In addition, the pharmacists only asked patients about the previous 7 days, whereas the PBS analyzed a 12-month period where use may be intermittent.

The lack of a change in adherence may seem surprising. In previous studies using similar interventions, we have observed an improvement in adherence (Saini et al., 2004; Axelsson and Lötvall, 2012; Armour et al., 2013; Serhal et al., 2021). For the interpretation of adherence, it may be that a different definition of “non-adherence” (80% PDC) would yield different results. The value of 80% is widely used in the literature, (Karve et al., 2009), but might not be relevant to all populations or healthcare systems. Using patient-reported adherence, results are consistent with the outcomes of previous studies, i.e., patients report improved adherence after an intervention (Armour et al., 2013). This suggests it is unreasonable to expect patients to have 80–100% adherence.

Despite this apparent lack of improvement in preventer medication use, the proportion of people with asthma who were using an inappropriate level of reliever medication was significantly reduced over the duration of the study in both the PAS and comparator arms. Inappropriate reliever overuse has been associated with tolerance, reduced benefit, increased risk of exacerbations and asthma death (Suissa et al., 1994; Aldridge et al., 2000; Haney and Hancox, 2005; Nwaru et al., 2020). On average, patients within the PAS arm reported using their reliever medication 15 times in the past 7 days; which equates to four cannisters per year. Overuse is defined as three or more cannisters of reliever medication per year and doubles the risk of exacerbations; therefore, the reduction we observed is likely to be clinically important (Suissa et al., 1994; Aldridge et al., 2000; Haney and Hancox, 2005; Nwaru et al., 2020).

The proportion of patients with device mastery at baseline is consistent with the published literature and what we would expect in a community sample of people with asthma (Sanchis et al., 2016). The almost doubling in proportion of patients who maintained device mastery at month 1 is consistent with inhaler device intervention studies (Basheti et al., 2009; Bosnic-Anticevich et al., 2010; Ovchinikova, 2014) and previous pharmacy asthma services research (Armour et al., 2013; Serhal et al., 2021). The fact that this increase in device mastery was sustained beyond the first month is an important finding. It indicates that the pharmacist intervention as it relates to inhaler technique is sustained over time for at least half the individuals who were not able to use their inhaler correctly at the start of the study. Future research and initiatives which lead to identifying the characteristics of patients at risk of not maintaining inhaler technique over time needs to build on preliminary research in this area (Ovchinikova, 2014). In so doing, pharmacists will have the potential to eliminate one of the most common barriers to poor asthma control in the community.

For allergic rhinitis, symptom control improved in both arms. In the PAS arm, pharmacists were required to undertake a detailed and structured assessment of symptoms and medication taking, using an evidence-based algorithm. Allergic rhinitis medication recommendations are part of routine clinical practice in pharmacy and may explain why allergic rhinitis symptom control improved in the comparator arm as well, where no structured intervention took place.

An earlier study had included an active referral to a GP for every asthma patient who did not have a current asthma action plan at baseline (Serhal et al., 2021). In the current study, this was not a recommendation until the final visit in both arms. When asked at the final visit, 39% of PAS patients and 50% of the comparator arm had an action plan. Given that the comparator arm received referral to the GP during the trial, it is likely that the GP initiated an asthma action plan where needed. Certainly, 50% is much higher than the proportion of the population with asthma in the Australian community who possess an action plan, which is approximately 28% (Australian Institute of Health and Welfare, 2018). Apart from a potential GP referral for an asthma action plan there were many opportunities flagged in the software for pharmacists to refer their patients to the GP. In addition, part of the comparator group protocol was a mandatory referral to a GP. Thus, the trial outcomes are a result of a complex interaction between the patient, their pharmacist and their GP. The investigative team did not separate these elements out. A partnership occurred but the role of each element may have been different for each patient depending on need.

What is made clear by the trial is that for a PAS to be efficacious and routinised into practice further research on an ideal level of service is needed. A balance is yet to be achieved between complexity, implementability and efficacy. In the past, more complex interventions were more effective but less implementable (Armour et al., 2007; Armour et al., 2013). The current PAS, which aimed to strip away the complexity of previous services and better integrate it into practice was effective, but more importantly, pharmacists appeared to be undertaking interventions as part of usual practice. Perhaps an ideal future model would draw upon differential service interest evident 20 years ago, which would see all pharmacies equipped to provide a minimal intervention service, and pharmacies choosing to specialize in respiratory conditions providing a more comprehensive service (Saini et al., 2001). Such a model would be likened to the provision of on-the-spot MedsChecks^3^ by all pharmacists but Home Medication Review^4^-accredited pharmacists provide an in-depth review based on specialized skills (Pharmaceutical Society of Australia, 2017).

### Limitations

The trial experienced a higher proportion of patient loss to follow up (33%), and subsequent pharmacy discontinuation (17%) than sample size calculations had allowed for (20 and 15%, respectively).

It is important when studying a sample of pharmacies that the results are generalizable. Participating pharmacies came from three states of Australia, were randomly allocated to either intervention or comparator arm and stratified according rurality to be representative of the spread of the Australian population. We thus believe we have a representative sample.

Recruitment ran over 7 months (Australian winter-Australian summer); as such, we cannot discount the effects of seasonality on asthma control, rhinitis control and medication use. However, any possible effects of seasons were the same for both arms. External climatic factors including dust storms and major bush fires which occurred during the trial are likely to have impacted negatively on patient control of their asthma. The air quality in many regions deteriorated during the bush fire season, and people with asthma were advised by health officials to stay indoors (Centre for Air Pollution, Energy and Health Research, 2019; Campbell et al., 2019; Rychetnik et al., 2019). The effects of these environmental disturbances on patient outcomes cannot be discounted, however, they are expected to be similar in both arms of the study as pharmacies were sampled from similar geographical areas.

## Conclusion

Comparable improvements in asthma control were experienced in both the PAS and minimal intervention, usual practice arm. However, the PAS was associated with greater improvements in patient quality of life. This research provides evidence that harnessing the skills of pharmacists and allowing them to contribute to their patients’ asthma management improves therapeutic and humanistic outcomes for patients. Further, it prompts reflection on current standards of usual care, as it appears the standard of asthma care in usual practice has evolved beyond that reported in the literature.

## Data Availability

The raw data supporting the conclusions of this article will be made available by the authors, without undue reservation.

## Footnotes

1 The MBS is a list of health services subsidized by the Australian Government. 34.

2 The PBS is an Australian Government initiative that subsidizes prescription medicines for Australian citizens. 35.

3 The MedsCheck service is an Australian Government funded in-pharmacy medication review for patients on multiple medications. Designed to promote quality use of medicines and reduce the number of consumer reported adverse effects (Health AGDo, 2014).

4 A Homes Medicines Review is an Australian Government funded at-home comprehensive medication review, designed to promote quality use of medicines. Home Medicines Reviews are conducted by accredited pharmacists on referral from a patients GP (Medication-adherence-and-medication-management-programs, 2015).

## Abbreviations

ACQ, Asthma Control Questionnaire; GP, General practitioners; IAQLQ, Impact of Asthma on Quality-of-Life Questionnaire; MBS, Medicare Benefits Schedule; NSW, New South Wales; PBS, Pharmaceutical Benefits Scheme; PhARIA, Pharmacy Access/Remoteness Index of Australia; pMDI, Pressurized metered-dose inhalers; PDC, Proportion of days covered; RCAT, Rhinitis Control Assessment Test; WA, Western Australia.
